# Feasibility of using volatile urine fingerprints for the differentiation of sexually transmitted infections

**DOI:** 10.1007/s00253-023-12711-0

**Published:** 2023-08-24

**Authors:** Ricardo Rubio-Sánchez, Cristina Ubeda, Rocío Ríos-Reina

**Affiliations:** 1 Servicio de Análisis Clínicos, Hospital Universitario Virgen de Valme, 41014 Seville, Spain; 2 Departamento de Nutrición y Bromatología, Toxicología y Medicina Legal, Facultad de Farmacia, Universidad de Sevilla, 41012 Seville, Spain

**Keywords:** Bacteria, Sexually transmitted infection, Urine, Vaginal swab, VOC, Volatile compounds

## Abstract

**Abstract:**

Sexually transmitted infections (STIs) are a public health problem worldwide, and current diagnostic methods have certain limitations. In recent years, volatile organic compounds (VOCs) have been studied as an alternative diagnostic method. Due to this, this study aimed to detect, in vaginal swabs and urine samples, VOCs emitted by highly prevalent STIs-causing bacteria (*Chlamydia trachomatis*, *Mycoplasma genitalium*, and *Neisseria gonorrhoeae*) to identify potential biomarkers that allow the detection of these STIs. VOCs detected in urine samples showed a better differentiation of patients with STIs due to *C. trachomatis* from those not infected, with 2,6-dimethyl-4-heptanone as the volatile compound most related to the presence of this bacterium. Among the VOCs most related to *M. genitalium* in urine, 4-methyltetradecane and 2-methylpentadecane stood out, while 3,4,4-trimethyl-2-cyclohexen-1-one was the VOC most closely related to *N. gonorrhoeae* infection. Moreover, C_12_ alcohols were the main VOC family associated with positive samples in all three bacteria, which could indicate the presence of aldehyde reductases in their metabolism. In contrast, alcohols such as 3-methyl-1-heptanol and 1-octanol, as well as dimethyl esters, were more associated with negative samples and may be useful in ruling out an STI caused by one of these three bacteria. In short, the VOCs identified as potential biomarkers in patients with infection by *C. trachomatis*, *M. genitalium*, or *N.* *gonorrhoeae* could be used in the early diagnosis of these STIs, quickly interrupting the chain of transmission, especially interesting in asymptomatic patients.

**Key points:**

*• Sexually transmitted infections are a serious public health problem worldwide.*

*• The study of VOCs in multiple infections is increasing in recent years.*

*• The identification of volatile biomarkers could allow new diagnostic methods.*

**Graphical abstract:**

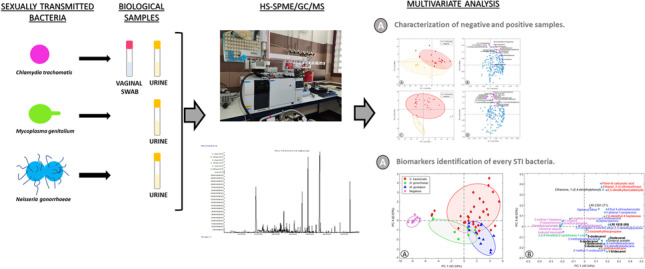

## Introduction

Sexually transmitted infections (STIs) are a public health problem worldwide since their incidence has increased significantly in recent years. According to the World Health Organization (WHO), 376 million new STIs occur annually, with *Chlamydia trachomatis*, *Neisseria gonorrhoeae*, *Trichomonas vaginalis*, and *Treponema pallidum* being the main etiological agents (Rowley et al. 2019). Some of the causes of this trend are the loss of fear of contracting HIV due to pre-exposure prophylaxis, the increase in risky sexual behaviors, the growing resistance to antibiotics, and the varied clinical presentation of these infections, with more than 50% of asymptomatic cases (Buder et al. 2019).

The suffered pathology is diverse and usually related to the genitourinary system. Thus, *C. trachomatis* serovars D-K cause urethritis in men and ascending infections leading to pelvic inflammatory disease in women (Morré et al. 2000), while serovars L1-L3 are associated with lymphogranuloma venereum (Lesiak-Markowicz et al. 2019). Manifestations of the *N. gonorrhoeae* infection are variable due to the considerable genetic variability that can cause urethritis with purulent discharge in men and increased vaginal discharge and dysuria in women (Buder et al. 2019). These two bacteria are the causes of most STIs worldwide, with an annual incidence of 34/1000 in *C. trachomatis* and 23/1000 in *N. gonorrhoeae* (Rowley et al. 2019). Furthermore, another STI bacterium, *Mycoplasma genitalium*, has an annual incidence of 11/1000 and causes urethritis with dysuria in men and cervicitis in women (Cina et al. 2019). The concern regarding *M. genitalium* is that STIs management guidelines do not clearly recommend screening for this bacterium, so it is not implemented in most microbiology laboratories (Treviño et al. 2021). Thus, many infections caused by *M. genitalium* are being treated with doses of azithromycin appropriate for *C. trachomatis* and *N. gonorrhoeae* but insufficient for *M.* *genitalium*, contributing to the development of macrolide resistance (Jensen et al. 2008, 2022). Infection by any of these three bacteria can cause serious complications such as pelvic inflammatory disease, infertility, and ectopic pregnancy. In addition, these microorganisms can also infect the rectum and pharynx, so the correct choice of the type of sample to make the diagnosis is essential (Rowley et al. 2019; Buder et al. 2019).

Therefore, the type of sample to collect for the diagnosis of an STI will depend on the patient’s sexual practices, medical history, and clinical symptoms. The sample most recommended and used for these STIs diagnosis in men is the urine from the first urination, while the specimen of choice in women is the vaginal swab. These samples have the advantage that they can be self-obtained, being much more comfortable for the patient (Jensen et al. 2022).

Traditional diagnostic methods, such as microscopy and culture, are time-consuming, can only be used on certain sample types, and may have low sensitivity and specificity (Karami et al. 2017). All of this is due to the fact that the main infectious agents involved in STIs are non-culturable, difficult to culture, or highly sensitive to transport and storage conditions, so techniques that only detect viable microorganisms can give false negative results, leaving transmission vectors without positive diagnosis and treatment (Galán et al. 2018).

Currently, nucleic acid amplification techniques (NAAT) are the reference method for STIs diagnosis because they have numerous advantages over traditional diagnostic techniques. NAATs detect non-viable microorganisms, thus increasing sensitivity and facilitating the collection, transport, and processing of more sample types. In addition, these techniques allow the simultaneous detection of multiple pathogens and establish the diagnosis in about 2 h (Buder et al. 2019). However, despite the generalization of NAATs against multiple targets, they have low sensitivity if samples are not handled correctly (Galán et al. 2018).

To solve this problem, in recent years, the volatile organic compounds (VOCs) emitted by microorganisms as part of their metabolism have been studied as an alternative diagnostic method. These compounds can be detected in body fluids by gas chromatography (GC) or high-performance liquid chromatography (HPLC) coupled with mass spectrometry (MS). Thus, GC–MS is considered the reference method for the isolation, identification, and quantification of VOCs (Karami et al. 2017; Kunze-Szikszay et al. 2021). Some volatile biomarkers have been identified in different infections, such as those produced by *Trichomonas vaginalis* (Rubio-Sánchez et al. 2023), *Listeria monocytogenes* (Lepe-Balsalobre et al. 2022), and *Mycobacterium tuberculosis* (Zetola et al. 2017), as well as in various diseases (i.e., Alzheimer’s disease (Ubeda et al. 2022), cancer (Wen et al. 2020), and diabetes mellitus (Esfahani et al. 2018)). However, to the best of our knowledge, there are no studies on VOCs produced in samples from STI patients. On the one hand, the vaginal metabolome of women with *C. trachomatis* and *M.* *genitalium* infection differs from that of women without STIs in the increase in biogenic amines, long-chain fatty acids, and fatty alcohols (Borgogna et al. 2020). On the other hand, increased sucrose, mannitol, pyruvate, lactate, and hippurate have been reported in the urine of women with *C. trachomatis* infection (Foschi et al. 2018; Gaspari et al. 2022).

In short, most STIs are asymptomatic and often go undiagnosed, so diagnosis should not only focus on symptomatic patients. Therefore, there is a need to implement new rapid and sensitive diagnostic methods that allow the detection and identification of microorganisms in biological fluids. The techniques that detect VOCs can be used as point-of-care-testing (POCT), so they allow a rapid diagnosis to be made at the patient’s care place, thus detecting asymptomatic reservoirs of the different STIs (Kunze-Szikszay et al. 2021). In this context, this study aimed to detect VOCs in vaginal swabs and/or urine from patients with infection by *Chlamydia trachomatis*, *Mycoplasma genitalium*, or *Neisseria gonorrhoeae* to identify potential biomarkers that allow the detection of these STIs and the ideal sample for diagnosis, identifying the causative bacterium.

## Materials and methods

### Samples

Two different types of samples (female vaginal swabs and male urine) were used in this study. Thirty-two vaginal swab specimens (22 positives for *C. trachomatis* and 10 negatives for this bacteria) and sixty urine samples (32 positives for *C. trachomatis*, 12 positives for *M. genitalium*, 6 positives for *N. gonorrhoeae*, and 10 negatives for these bacteria) from different patients to avoid cross-contamination of VOCs were collected. In addition, 3 vaginal swab transport media without biological samples were also included in the study. The mean age of women was 29.6 years (range: 19–65), while the mean age of men was 30.3 years (range: 18–51).

Vaginal swab specimens were collected and transported with an Aptima vaginal swab specimen collection kit (Hologic, USA) containing 2.9 mL of transport medium, while urine samples were collected and transported with Aptima urine specimen transport tubes (Hologic, USA) containing 2 mL of transport medium. Detection of *C. trachomatis*, *M.* genitalium, and N*. gonorrhoeae* in vaginal swabs and urine specimens was performed using the Aptima assay on the Panther system.

After analyzing and classifying the samples according to the Aptima assay results, 2 mL of transport medium with vaginal swab specimen or urine were placed in a 20-mL headspace vial and frozen at − 80 °C until the VOCs were analyzed.

This study was performed in line with the principles of the Declaration of Helsinki. Approval was granted by the Clinical Research Ethics Committee Ntra. Sra. de Valme University Hospital (Code: 0170-N-20).

### Chemicals

A standard mixture of C_10_–C_40_ alkanes, supplied by Fluka (Madrid, Spain), was used to calculate the linear retention index (LRI). In addition, 4-methyl-2-pentanol (internal standard), sodium chloride, and ethanol were supplied by Merck (Darmstadt, Germany).

### Headspace-solid phase microextraction (HS-SPME)

The headspace vials with the samples were thawed at 4 °C for 12 h, and then 0.5 g of sodium chloride and 10 µL of 4-methyl-2-pentanol (0.75 mg/L in milli Q water) were added. The extraction of the volatile compounds was carried out by means of HS-SPME. To do this, an MPS autosampler (Gerstel, USA) incubated the vial for 5 min at 45 °C with shaking at 300 rpm. Then, a 2 cm 50/30 µm Carboxen/DVB/PDMS SPME fiber (Supelco, USA) was exposed to the headspace of the vial for 30 min. The fiber was then desorbed in the injector in a splitless mode for 180 s with the transfer line at 250 °C.

### Gas chromatography–mass spectrometry analysis

Analyses were performed on an Agilent 8890 gas chromatograph coupled to an Agilent 5977B Inert Plus quadrupole mass spectrometer with a Gerstel autosampler (Müllheim an der Ruhr, Germany). The capillary column used was a J&W CPWax-57CB of 50 m × 0.25 mm and 0.20 µm film thickness (Agilent, USA), and the helium flow rate was 1 mL/min. The oven started at 35 °C held for 4 min, followed by an increase to 220 °C at 2.5 °C/min held for 1 min. Electron ionization mass spectral data were recorded from m/z 29–300 in scan mode with an ionization voltage of 70 eV.

### Data treatment and chemometrics

Chromatographic data were exported to AIA format using MSD ChemStation software (Agilent Technologies, USA). Subsequently, these data were processed using the Deconvolution and Identification System, known as PARADISe software. This software allows peak deconvolution by PARAllel FACtor analysis2 (PARAFAC2) modelling, and, simultaneously, it extracts the pure spectra of co-eluting compounds used for peak identification by comparing it with the reference from the NIST MS library (National Institute of Standards and Technology). In this case, modelling options were set to a maximum of 7 components per interval, non-negativity constrain was applied, and fit and core consistency were carefully optimized for selecting the correct number of components for each model. Finally, it generates an identification report with the areas of the different resolved peaks (Johnsen et al. 2017).

Moreover, identification was confirmed by comparing the LRI of standards with data from the literature. The LRIs were calculated using the retention times of a series of n-alkanes analyzed under the same conditions as the samples. The data shown in this work were expressed as a relative area concerning 4-methyl-2-pentanol (internal standard).

Once the relative areas of each dataset were obtained, they were subjected to an unsupervised analysis using Principal Component Analysis (PCA) to explore the grouping and differentiation of the samples. Different PCA models were developed: first, PCA models were created including only the dataset of a specific bacterium versus the negative samples; then, a PCA model was developed with the total of samples grouping them into positive versus negatives; and finally, two PCA models were created with the dataset of all the samples, including only the selected compounds as potential biomarkers of each class, one only for the positive samples and another one that includes the negative ones.

In addition, partial least squares discriminant analysis (PLS-DA) models were developed with the same datasets as with PCA, i.e., with each type of sample, each bacterium studied, and the total of VOCs, to carry out a data reduction and compound selection strategy based on the selection of variables with importance in the projection (VIPs). Hence, PLS-DA was not developed directly for a classification approach but was applied to identify the VOCs that allow differentiation between patients infected with any of the three sexually transmitted bacteria and patients without these STIs. PLS-DA models were cross-validated (CV) by venetian blinds, and the appropriate number of latent variables were selected according to the minimum CV classification error average. According to the literature (Mehmood et al. 2012), all predictors having a VIP > 1 are considered relevant. However, in this study, to greatly reduce the large number of compounds obtained and to search for specific biomarkers for each infection, variables whose VIP in each of the PLS-DA models were equal or greater than 2 (VIPs ≥ 2) were highlighted and sorted from highest to lowest VIP value since the higher the VIP score, the higher its contribution in the classification. PCA and PLS-DA were conducted using PLS Toolbox 7.9.5 (Eigenvector Research Inc., USA), working in a MATLAB 2016a environment (Mathworks). Before any modelling, the data were autoscaled.

## Results

PARADISe software was used to process two global GC–MS datasets: one formed by 32 vaginal swab samples and the other with 60 urine samples. Many VOCs were deconvoluted and tentatively identified in the individual datasets: 267 in vaginal swabs and 193 in urine specimens. Once the two datasets consisting of peak areas and VOC identification were obtained, and the relative areas calculated, they were divided into several subsets and were submitted to modelling for different purposes.

### Volatile biomarkers of the presence of *Chlamydia trachomatis* in vaginal swab and urine

*C. trachomatis* is the bacterium that causes STIs with the highest annual incidence worldwide, so we decided to study the VOCs present in the samples most used in this STI diagnosis, that is, vaginal exudate in women and urine in men. For this, two PCA models were developed with vaginal swabs data (22 × 267) and urine data (32 × 193).

**Fig. 1:**
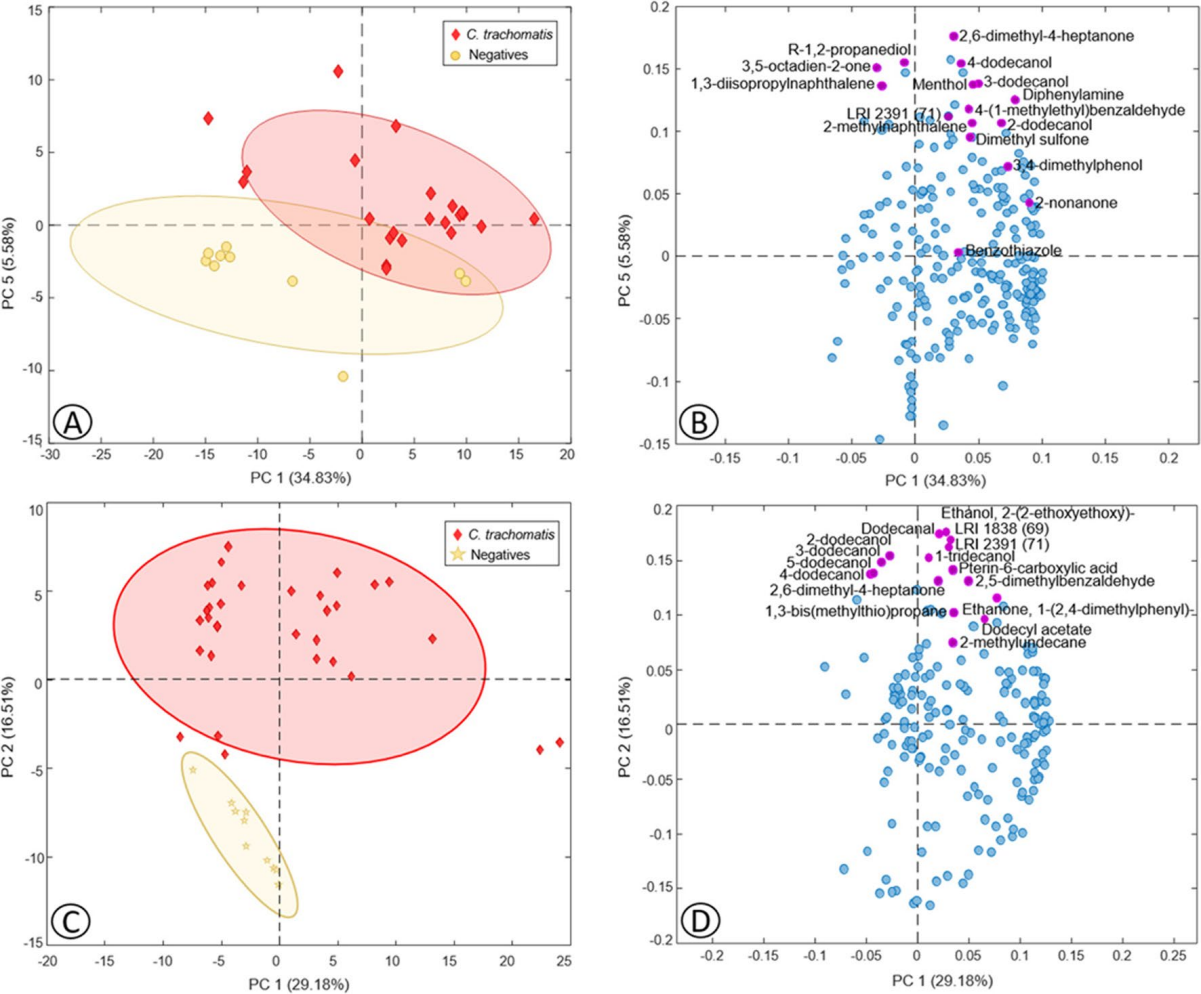
Scores (**A**, **C**) and loading (**B**, **D**) plots of two PCs obtained by PCA carried out on the vaginal swab (**A**, **B**) and urine (**C**, **D**) datasets with the total number of VOCs detected in *Chlamydia trachomatis*

Regarding the vaginal swab specimens, Fig. 1 shows the PCA scores (Fig. 1A) and loadings (Fig. 1B) plots displaying two principal components (PCs) obtained with all the VOCs and the negative and positive samples in *C. trachomatis*. Grouping of the samples according to the presence or absence of *C. trachomatis* could be seen (Fig. 1A). The loadings plot (Fig. 1B) shows 16 VOCs highlighted in purple that were those with VIP ≥ 2 and more related to positive samples (highlighted in boldface in Table 1), obtained by a 2-latent variable (LV) PLS-DA model carried out with the same dataset and developed to search for the most relevant volatile compounds in the differentiation of *C. trachomatis* from negative samples in vaginal swab dataset. Sensitivity and specificity in PLS-DA calibration and cross-validation were > 90% and an error rate of < 7%. The relative area ranges and LRI of these 16 selected VOCs are shown in Table 1 highlighted in boldface, being most of them from alcohol family (6) and benzene compounds (4) (Table 1).

**Table 1:** Relative area ranges and linear retention index (LRI) of the volatile organic compounds (VOCs) selected according to the VIP scores (≥ 2), highlighted for every bacteria in boldface, and their higher relation with positive samples obtained by PLS-DA models in vaginal swab and urine datasets

VOCs with VIP ≥ 2	LRI	Id	Vaginal swab		Urine				Transport medium
			*Chlamydia trachomatis*	Negative	*Chlamydia trachomatis*	*Mycoplasma genitalium*	*Neisseria gonorrhoeae*	Negative	
Alkanes									
2-Methylundecane	1154	A	0.28–1.04	0.42–0.78	**0.99–2.66**	1.09–2.65	1.27–2.02	0.38–1.69	0.91
4,6-Dimethyldodecane	1281	B	0.28–1.19	0.46–1.05	0.34–1.98	**0.60–2.33**	0.42–1.36	0.16–0.78	1.04
4-Methyltetradecane	1411	A	0.07–0.45	0.10–0.43	0.19–1.31	**0.45–1.70**	0.23–0.92	0.17–0.45	0.15
2-Methylpentadecane	1557	B	0.00–1.12	0.11–0.32	0.11–1.05	**0.31–1.22**	0.11–0.62	0.12–0.29	0.24
Ketones									
2,6-Dimethyl-4-heptanone	1164	A	**0.57–0.90**	0.67–0.72	**0.91–1.18**	0.91–1.05	0.84–0.97	0.00–0.76	0.74
2-Nonanone	1381	A	**0.36–0.71**	0.23–0.49	0.30–2.16	0.34–0.70	0.18–0.86	0.07–0.65	0.59
3,5-Octadien-2-one	1517	A	**0.00–0.06**	0.00–0.02	0.05–0.58	**0.13–0.47**	0.05–0.25	0.06–0.13	0.06
2-Undecanone	1595	A	0.12–0.45	0.07–0.35	0.10–0.66	**0.20–0.55**	0.09–0.39	0.16–0.25	0.42
1-Phenyl-1-propanone	1721	A	0.00–0.36	0.08–0.24	0.06–0.26	**0.13–0.19**	0.07–0.17	0.03–0.11	0.21
3,4,4-trimethyl-2-cyclohexen-1-one	1736	A	0.36–2.68	0.71–1.18	0.95–2.72	1.06–2.20	**2.01–2.93**	0.71–1.74	0,90
Ethanone, 1-(2,4-dimethylphenyl)-	2229	C	0.10–0.29	0.10–0.20	**0.10–0.38**	0.24–0.24	**0.13–0.22**	0.05–0.11	0.17
Alcohols									
R-1,2-propanediol	1610	A	**0.00–0.16**	0.00–0.01	ND	ND	ND	ND	ND
Ethanol, 2-(2-ethoxyethoxy)-	1634	A	0.14–0.31	0.22–0.33	**0.25–0.43**	0.23–0.32	0.24–0.27	0.21–0.26	0.22
Menthol	1650	A	**0.01–0.12**	0.00–0.04	0.05–1.81	0.09–11.74	0.05–0.33	0.07–4.75	0.02
5-Dodecanol	1785	A	0.20–1.55	0.06–0.48	**0.02–1.41**	**0.23–0.64**	**0.03–0.75**	0.02–0.05	0.47
4-Dodecanol	1786	A	**0.14–1.14**	0.03–0.37	**0.00–2.29**	**0.35–1.02**	**0.03–1.20**	0.00–0.05	0,20
3-Dodecanol	1801	A	**0.11–1.79**	0.00–0.66	**0.11–3.46**	**0.74–1.86**	**0.15–1.80**	0.09–0.17	0.43
2-Dodecanol	1830	A	**0.87–4.18**	0.09–2.81	**0.09–7.26**	**1.47–4.14**	**0.16–3.52**	0.07–0.29	2.01
2-Methyl-1-undecanol	2014	B	0.11–0.55	0.00–0.44	0.00–0.58	**0.08–0.62**	0.00–0.35	0.00–0.01	0.11
1-Tridecanol	2085	A	0.06–0.32	0.04–0.28	**0.00–0.36**	**0.13–0.26**	**0.02–0.15**	0.00–0.04	0.33
3,4-Dimethylphenol	2223	A	**0.03**–**0.10**	0.03–0.06	ND	ND	ND	ND	0.08
Aldehydes									
2,5-Dimethylbenzaldehyde	1704	A	ND	ND	**0.09–0.45**	0.11–0.26	0.14–0.22	0.06–0.18	ND
Dodecanal	1708	A	2.76–6.61	1.34–5.96	**0.66–4.51**	**2.79–5.38**	**1.27–3.22**	0.67–1.63	6.60
4-(1-Methylethyl)benzaldehyde	1823	A	**0.33–0.78**	0.29–0.53	0.28–1.71	0.38–0.71	0.30–0.52	0.32–0.87	0,50
Benzene compounds									
2-Methylnaphthalene	1844	A	**0.05–0.15**	0.04–0.10	0.03–0.22	0.05–0.12	0.07–0.16	0.06–0.12	0.19
Benzothiazole	1966	A	**0.04–0.20**	0.02–0.05	ND	ND	ND	ND	0.03
1,3-Diisopropylnaphthalene	2143	A	**0.00–0.10**	0.00–0.01	ND	ND	ND	ND	ND
Diphenylamine	2434	C	**1.33–1.93**	1.09–1.42	0.02–0.50	**0.11–0.26**	0.03–0.16	0.04–0.06	2.14
Esters									
Dodecyl acetate	1886	A	0.11–0.43	0.05–0.35	**0.06–0.66**	**0.21–0.70**	0.11–0.42	0.11–0.37	0.41
Ethyl 4-ethoxybenzoate	2179	C	0.64–1.97	0.29–1.99	0.13–7.09	**0.60–2.75**	0.21–2.30	0.33–1.21	5,90
Miscellanea									
5-Ethyl-2,3-dimethylpyrazine	1469	A	0.17–1.55	0.45–1.22	0.01–0.72	**0.13–0.59**	0.03–0.29	0.03–0.13	1.39
1,3-Bis(methylthio)propane	1573	A	ND	ND	**0.00–0.01**	0.00–2.40	ND	ND	ND
Dimethyl sulfone	1915	A	**0.04–0.14**	0.02–0.08	0.02–0.20	0.04–0.20	0.03–0.27	0.03–0.08	0.06
Pterin-6-carboxylic acid	1960	B	0.06–0.13	0.10–0.23	**0.14–0.37**	0.12–0.24	0.10–0.13	0.11–0.15	0.21
Diphenyl ether	2007	A	ND	ND	0.01–0.73	**0.02–0.05**	0.01–0.03	0.01–0.01	ND
Non identified									
69, 83, 85	1838	–	0.15–0.49	0.10–0.40	**0.07–0.87**	**0.36–0.75**	**0.13–0.40**	0.05–0.15	0.42
71, 55, 73	2391	–	**0.10–0.92**	0.15–0.72	**0.26–0.87**	**0.29–0.65**	0.24–0.52	0.06–0.29	0.48

The identification of VOCs was carried out by comparing each mass spectra obtained with mass spectral data base (NIST) and LRI agreed with data from the literature (Pherobase: www.pherobase.com, NIST Mass Spectrometry Data Center: https://webbook.nist.gov, LRI and Odour database: http://www.odour.org.uk/lriindex.html). *ND*, non detected. Data expressed as relative areas are multiplied by 100. *Id*, identification reliability. *A*, mass spectrum agreed with mass spectral data base and LRI agreed with the literature data. *B*, mass spectrum agreed with mass spectral data base, but there is no LRI in a polar column reported in the literature. *C*, mass spectrum agreed with mass spectral data base RMatch ˃ 800 but not with LRI in the literature

Regarding urine VOC dataset, Fig. 1 also shows the PCA scores (Fig. 1C) and loadings (Fig. 1D) plots displaying the first two PCs where the separation of the samples according to the presence of *C. trachomatis* from those negatives in the urine can be observed mainly through PC2 (Fig. 1C). Once again, the loadings plot (Fig. 1D) shows 16 VOCs with VIPs ≥ 2 obtained by a 2-LV PLS-DA model made with the same urine dataset and highlighted in purple for being more related to positive samples. Sensitivity and specificity of this model were > 93% and an error rate of < 3%, providing slightly better results than the model made with vaginal swab. Among these 16 highlighted compounds, many of them were alcohols (6), ketones (2), and aldehydes (2) (Table 1).

### Volatile biomarkers of the presence of other STI-causing bacteria in urine

As can be seen from the above results, the volatile profile of urine samples was more effective than the vaginal swab volatile profile in distinguishing patients infected with *C. trachomatis* from those without this STI. Thus, PCA scores plot of the urine dataset (Fig. 1C) showed better separation of negative and positive samples, and classification results were also slightly better than in vaginal swabs. Therefore, given these promising results, urine VOCs from patients infected with two other highly prevalent STI-causing bacteria (*M. genitalium* and *N.* *gonorrhoeae*) were analyzed for potential biomarkers of their presence in urine.

#### Volatile biomarkers of the presence of *Mycoplasma genitalium* in urine

**Fig. 2:**
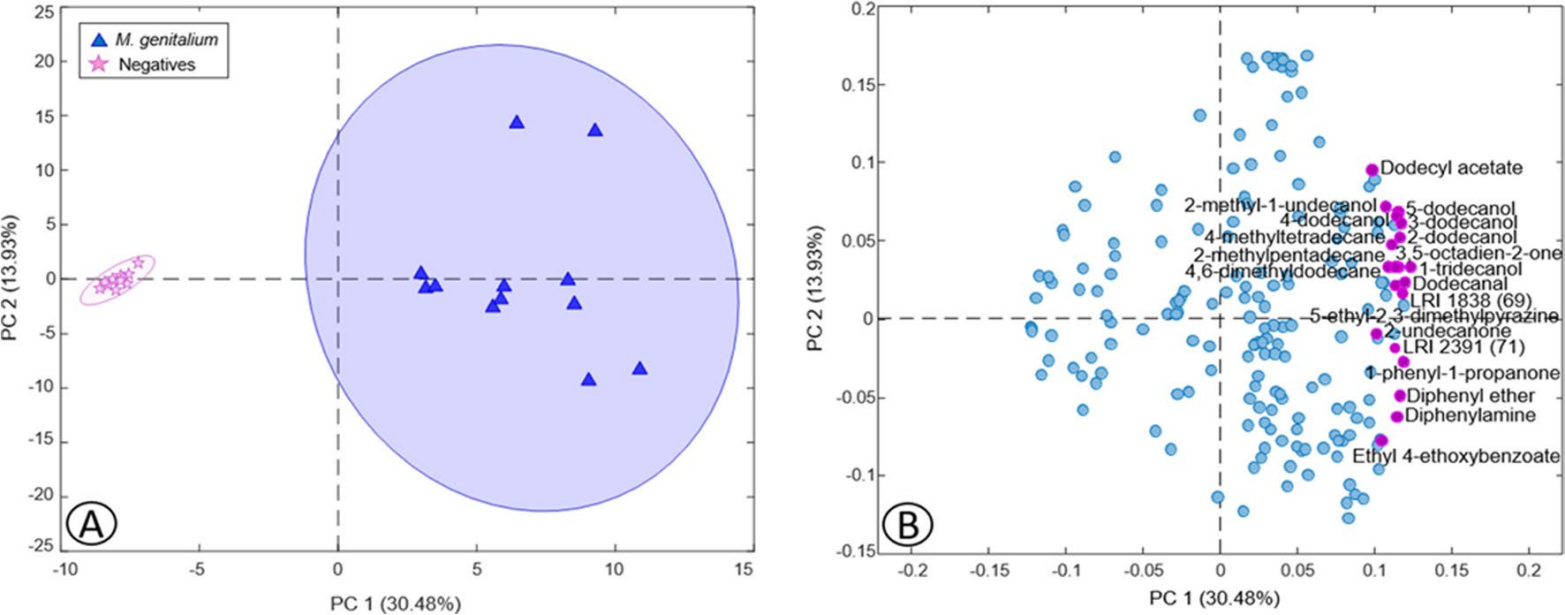
Scores (**A**) and loadings (**B**) plots of two PCs obtained by PCA based on the urine dataset with the total number of VOCs detected in *Mycoplasma genitalium*

A PCA model was developed with the urine dataset (22 × 193) containing the total of VOCs identified in the negative and positive *M.* *genitalium* samples. Figure 2 shows the PCA scores (Fig. 2A) and loadings (Fig. 2B) plots displaying the first two PCs where a clear differentiation between positive and negative samples can be observed by their position in the positive and negative side of PC1, respectively (Fig. 2A). As was displayed in the PCA of *C. trachomatis* (Fig. 1), the 20 VOCs with VIPs ≥ 2 more related to *M.* *genitalium* samples were highlighted in purple in the loadings plot (Fig. 2B) and in boldface in Table 1. These VIPs were obtained by a 2-LV PLS-DA model developed with the same urine dataset, with a sensitivity and specificity in calibration and CV of 100%, as was expected according to the PCA scores plot.

#### Volatile biomarkers of the presence of *Neisseria gonorrhoeae* in urine

**Fig. 3:**
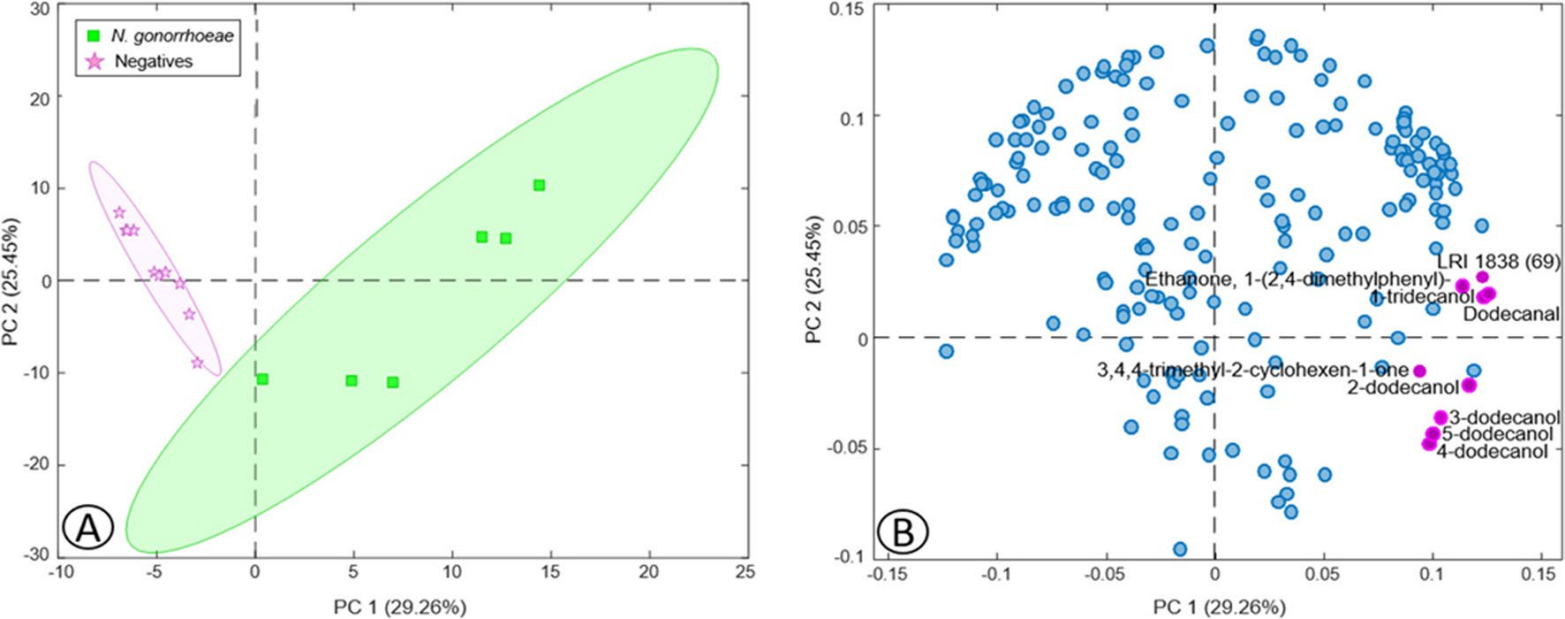
Scores (**A**) and loadings (**B**) plots of two PCs obtained by PCA based on urine dataset with the total number of VOCs detected in *Neisseria gonorrhoeae*

Another PCA model was carried out with the dataset formed by negative and positive *N.* *gonorrhoeae* urine samples and the total of VOCs identified in the global urine dataset (16 × 193). Figure 3 shows the PCA scores (A) and loadings (B) plots displaying the first two PCs, where the separation of samples with the presence of *N.* *gonorrhoeae* from the negative ones in urine can be clearly observed mainly by PC1 (Fig. 3A). In addition, a 3-LV PLS-DA model was developed with this dataset, and from the total of VOCs with VIPs values ≥ 2, 9 were more related to positive samples and highlighted in purple in the loadings plot (Fig. 3B) and in boldface in Table 1. In this case, this model also showed sensitivity and specificity in calibration and CV of 100%, as occurs with *M.* *genitalium* urine dataset.

### Specific volatile biomarkers for the differentiation of the STI-causing bacteria in urine

**Fig. 4:**
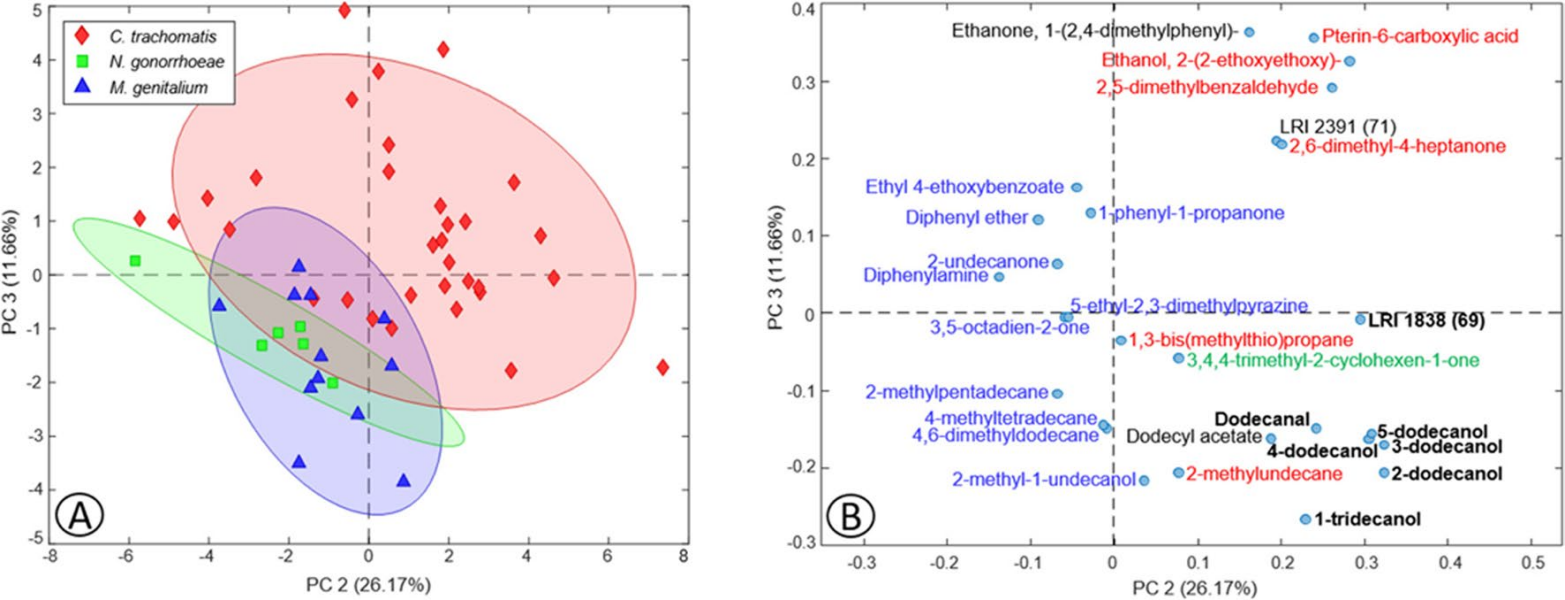
Scores (**A**) and loadings (**B**) plots of two PCs obtained by PCA based on urine dataset with all VIPs ≥ 2 previously identified. Loadings are colored according to the colors of each sample type in the scores plot

As it was shown, several of the VOCs related to the presence of some of the three STI bacteria were common in positive urine samples. Due to this, the next objective was to verify which were characteristic VOCs of each bacterium, i.e., that allowed the differentiation of an STI bacteria from another. For that, a PCA model was developed by including the positive samples and the 28 VOCs with VIP ≥ 2 that were more related to each bacterium, obtained in the three previous individual PLS-DA models (Fig. 4). Thus, 6, 11, and 1 VOCs were included as the specific VIP VOCs of *C. trachomatis*, *M.* *genitalium*, and *N. gonorrhoeae*, respectively, and were colored according to them in the PCA loadings plot (Fig. 4B). In addition, 3 VOCs that were common to two bacteria, colored in black in the loadings plot, and another 7 VOCs that were common to all three bacteria, marked in bold, were included in the PCA model.

As can be seen in the scores plot (Fig. 4A), VOCs with VIPS ≥ 2 previously identified allowed samples to be grouped according to each bacterium, although with some overlap that could be due to the presence of common VOCs. Thus, these VOCs that were not common among bacteria could be considered potential biomarkers of the specific presence of some of the bacteria that cause STIs in urine. In the case of *C. trachomatis*, the urinary VOCs with the greatest potential to be biomarkers would be 2,6-dimethyl-4-heptanone, ethanol, 2-(2-ethoxyethoxy), and 2,5-dimethylbenzaldehyde (colored in red in Fig. 4B). In contrast, in patients with *M. genitalium* infection, the potential volatile biomarkers in urine were 4-methyltetradecane and 2-methylpentadecane (colored in blue), while in *N.* *gonorrhoeae* infection was 3,4,4-trimethyl-2-cyclohexen-1-one (colored in green). This report is the first to point to these urinary VOCs as possible volatile biomarkers of sexually transmitted infections caused by these bacteria. These results confirm that the 28 VOCs previously identified could be potential volatile biomarkers of the presence of an STI, as well as of a specific STI-causing bacterium.

## Discussion

As far as we know, this is the first time PARADISe has been applied to these types of samples. Its application has the advantage of performing an easy and fast integration and identification of VOCs from a large set of samples. This software is also robust since it allows the correction of the baseline and noise, being very useful in the identification of minor compounds in complex volatile profiles (Ríos-Reina et al. 2023).

### Volatile biomarkers of the presence of *Chlamydia trachomatis* in vaginal swab and urine

Diphenylamine was the VOC with the highest VIP score in vaginal swab (VIP = 4.62) (Table 2). As could be observed, this compound showed a higher presence in the transport medium (Table 1), and, according to the literature, it is degraded by bacteria and fungi (Perruchon et al. 2015), agreeing this with the present results. However, results pointed out that diphenylamine was not as preferred by *C. trachomatis* as its carbon and nitrogen source as other compounds but seemed to be preferred by the predominant bacteria in vaginal swabs from patients without this STI, as the relative area values in negative swab samples were lower than in the positive ones for this bacterium. This could be explained by the fact that patients with *C. trachomatis* infection have a different vaginal metabolome, mainly based on carbohydrates and amino acids, compared with patients without this STI, as has been recently found (Borgogna et al. 2020).

**Table 2:** Score values of the variable importance in projection (VIP) index obtained by the PLS-DA classification model made with vaginal swab dataset

VOCs with VIP ≥ 2 in vaginal swab	Chemical family	VIP scores	More related to
*Chlamydia trachomatis*			
Diphenylamine	Benzene compounds	4.62	P
2,6-Dimethyl-4-heptanone	Ketones	4.31	P
3,5-Octadien-2-one	Ketones	4.05	P
LRI 2391 (71)	–	3.67	P
Dimethyl sulfone	Sulfur compounds	3.60	P
3,4-Dimethylphenol	Alcohols	3.29	P
R-1,2-propanediol	Alcohols	3.27	P
4-(1-Methylethyl)benzaldehyde	Aldehydes	2.86	P
Benzothiazole	Benzene compounds	2.69	P
2-Nonanone	Ketones	2.55	P
Menthol	Alcohols	2.50	P
2-Dodecanol	Alcohols	2.49	P
3-Dodecanol	Alcohols	2.49	P
1,3-Diisopropylnaphthalene	Benzene compounds	2.32	P
4-Dodecanol	Alcohols	2.22	P
2-Methylnaphthalene	Benzene compounds	2.05	P
Pterin-6-carboxylic acid	Acids	6.80	N
4-Propylbenzaldehyde	Aldehydes	4.44	N
3-Methyl-1-pentanol	Alcohols	3.86	N
Methyl dihydrojasmonate	Esters	2.57	N

*P* positive samples (infected people),* N* negative or control samples (healthy people)

Another similar case was the occurrence of dimethyl sulfone, another VIP ≥ 2 in vaginal swab more presented in positive *C. trachomatis* samples, which was not so relevant in the differentiation among positive and negative urine samples (Table 1). Sulfur compounds are typical metabolites from microbial activity, and, concretely, dimethyl sulfone has been found in fecal and urine human samples as a derivative compound from the metabolism of methionine by the microbiota (He and Slupsky 2014), which may explain its relevant presence in *C. trachomatis* positive samples.

Instead, 2,6-dimethyl-4-heptanone was the compound with the highest VIP score and related to positive *C. trachomatis* samples in urine (VIP = 5.05), being also this ketone the second with the highest VIP scores in positive vaginal swab samples (Tables 2 and 3). Although this compound is produced by different species of *Lactobacillus* (Di Renzo et al. 2018) that can be found in the vagina (Zhang et al. 2012), according to our results, the highest production of 2,6-dimethyl-4-heptanone occurs in samples with *C. trachomatis*, especially in urine (Table 1). Hence, five VOCs with VIPs ≥ 2 related to positive samples were common between the two types of biological samples (2,6-dimethyl-4-heptanone, 4-dodecanol, 3-dodecanol, 2-dodecanol, and no-identified compound (LRI 2391, 71)) (Tables 2 and 3), so the increase of these VOCs in vaginal swab and urine could be related to the presence of *C. trachomatis*.

**Table 3:** Score values of the variable importance in projection (VIP) index obtained by the PLS-DA classification model made with urine dataset

VOCs with VIP ≥ 2 in urine	Chemical family	VIP values from individual PLS-DA models	VIP values from global PLS-DA model	More related to
*Chlamydia trachomatis*				
2,6-Dimethyl-4-heptanone	Ketones	5.05	4.80	P
LRI 2391 (71)	–	4.79	3.92	P
Ethanone, 1-(2,4-dimethylphenyl)-	Ketones	4.11	2.64	P
Fodecanal	Aldehydes	3.85	–	P
Ethanol, 2-(2-ethoxyethoxy)-	Alcohols	3.82	–	P
LRI 1838 (69)	–	3.62	3.06	P
2-Dodecanol	Alcohols	3.21	2.98	P
Pterin-6-carboxylic acid	Acids	3.07	–	P
3-Dodecanol	Alcohols	3.02	2.64	P
2,5-Dimethylbenzaldehyde	Aldehydes	2.85	–	P
1-Tridecanol	Alcohols	2.82	2.84	P
5-Dodecanol	Alcohols	2.60	2.15	P
4-Dodecanol	Alcohols	2.51	2.15	P
1,3-Bis(methylthio)propane	Sulfur compounds	2.32	–	P
2-Methylundecane	Ketones	2.25	–	P
Dodecyl acetate	Esters	2.22	–	P
Dimethyl adipate	Esters	6.74	9.93	N
Dimethyl glutarate	Esters	6.47	8.96	N
Isobutyl benzoate	Esters	5.13	6.06	N
Propiophenone	Ketones	4.68	6.40	N
1-Octanol	Alcohols	4.52	5.98	N
3-Methyl-1-heptanol	Alcohols	4.49	5.52	N
6-Pentadecanol	Alcohols	4.41	5.38	N
Dimethyl succinate	Esters	4.28	4.79	N
6-Methyl-1-heptanol	Alcohols	2.91	3.86	N
*Mycoplasma genitalium*				
1-Tridecanol	Alcohols	3.05	2.84	P
4-Dodecanol	Alcohols	2.93	2.15	P
2-Dodecanol	Alcohols	2.91	2.98	P
3-Dodecanol	Alcohols	2.90	2.64	P
5-Dodecanol	Alcohols	2.87	2.15	P
LRI 1838 (69)	–	2.82	3.06	P
Dodecanal	Aldehydes	2.82	–	P
1-Phenyl-1-propanone	Ketones	2.73	–	P
2-Methyl-1-undecanol	Alcohols	2.64	–	P
LRI 2391 (71)	–	2.54	3.92	P
Diphenyl ether	Ethers	2.31	–	P
Diphenylamine	Benzene compounds	2.28	–	P
3,5-Octadien-2-one	Ketones	2.25	–	P
2-Undecanone	Ketones	2.24	–	P
2-Methylpentadecane	Alkanes	2.23	–	P
4-Methyltetradecane	Alkanes	2.15	–	P
5-Ethyl-2,3-dimethylpyrazine	Nitrogen compounds	2.14	–	P
Ethyl 4-ethoxybenzoate	Esters	2.11	–	P
Dodecyl acetate	Esters	2.07	–	P
4,6-Dimethyldodecane	Alkanes	2.06	–	P
Dimethyl succinate	Esters	3.21	4.79	N
Dimethyl glutarate	Esters	3.16	8.96	N
Dimethyl adipate	Esters	3.12	9.93	N
6-Pentadecanol	Alcohols	2.91	5.38	N
3-Methyl-1-heptanol	Alcohols	2.81	5.52	N
Isobutyl benzoate	Esters	2.56	6.06	N
Propiophenone	Ketones	2.53	6.40	N
1-Octanol	Alcohols	2.40	5.98	N
6-Methyl-1-heptanol	Alcohols	2.26	3.86	N
*Neisseria gonorrhoeae*				
3,4,4-Trimethyl-2-cyclohexen-1-one	Ketones	3.10	–	P
Ethanone, 1-(2,4-dimethylphenyl)-	Ketones	2.84	2.64	P
2-Dodecanol	Alcohols	2.64	2.98	P
Dodecanal	Aldehydes	2.53	–	P
1-Tridecanol	Alcohols	2.51	2.84	P
3-Dodecanol	Alcohols	2.48	2.64	P
LRI 1838 (69)	–	2.45	3.06	P
4-Dodecanol	Alcohols	2.28	2.15	P
5-Dodecanol	Alcohols	2.28	2.15	P
Dimethyl adipate	Esters	3.19	9.93	N
Dimethyl glutarate	Esters	3.13	8.96	N
6-Pentadecanol	Alcohols	2.79	5.38	N
3-Methyl-1-heptanol	Alcohols	2.73	5.52	N
1-Octanol	Alcohols	2.69	5.98	N
6-Methyl-1-heptanol	Alcohols	2.61	3.86	N
Isobutyl benzoate	Esters	2.55	6.06	N
Propiophenone	Ketones	2.42	6.40	N
Dimethyl succinate	Esters	2.34	4.79	N

*P* positive samples (infected people),* N* negative or control samples (healthy people)

Another compound found to be relevant in urine samples from patients with *C. trachomatis* was pterin-6-carboxylic acid (Table 1). This acid has been described as a metabolite of folic acid degradation by *Pseudomonas aeruginosa* (Bacher and Rappold 1980). Due to the renal excretion of folic acid and its presence in urine (Goresky et al. 1963), it could be metabolized by *C. trachomatis*, increasing the content of pterin-6-carboxylic acid in the urine of patients with this infection.

Moreover, among the VOCs with VIP ≥ 2 related to the presence of this bacterium, both in vaginal swabs and urine, several volatile compounds formed by a 12-carbon chain were observed (Table 1). In the case of vaginal swab samples, specifically, a remarkable amount of C_12_ alcohols was strongly related to the presence of *C. trachomatis*, such as 2-dodecanol, 3-dodecanol, 4-dodecanol, and 5-dodecanol. Considering the high amount of dodecanal found in the transport medium, it could be happening a reduction of this aldehyde to the derivative alcohols by the bacteria reductases since the presence of dodecanal decreases in the samples with the bacteria. In the case of urine, it can be also observed the decrease of dodecanal and the increment of C_12_ alcohols. Results also revealed that the microbiota of the urines without *C. trachomatis* also carry out a high degradation of dodecanal; however, the reduction pathway to C_12_ alcohols does not occur (Table 1). It has been described that dodecanol produced by the fungus *Conidiobolus coronatus* helps this microorganism to infect some insects and has been proposed as a good insecticide candidate (Kazek et al. 2021). It has also been claimed that dodecanol has anti-*Salmonella* activity (Fujita et al. 2015) and that it can inhibit the filamentation of *Candida albicans* (Hogan et al. 2004). Therefore, *C. trachomatis* could be producing dodecanol as a possible strategy/mechanism to facilitate the infection.

In addition, as can be seen in the scores plots of vaginal swab and urine PCA models (Fig. 1A and C, respectively), negative urine samples are more homogeneous and had less variation between patients than in the vaginal swab. It must be considered that the healthy vaginal microbiota is dominated by *Lactobacillus* spp., which produces lactic acid that lows vaginal pH, making this acidic environment inhospitable to many vaginal pathogens. In addition, these bacteria also produce antibacterial compounds and act as a mechanical barrier, protecting the host from STI-causing bacteria. When vaginal dysbiosis occurs, these protective mechanisms disappear, biogenic amines are synthesized, and susceptibility to STIs increases (Nelson et al. 2015; Molenaar et al. 2018). Therefore, that heterogeneity of negative vaginal swab samples could be since some women without STIs had bacterial vaginosis, which usually presents fewer vaginal microbial communities of *Lactobacillus* spp., but a high variety of anaerobes (Borgogna et al. 2020). In contrast, negative urine samples used in this study did not show bacteriuria and therefore did not contain microorganisms. Due to this, the greater dispersion in vaginal swab samples would demonstrate the capacity of the bacteria to generate VOCs as part of their metabolism.

### Volatile biomarkers of the presence of *Mycoplasma genitalium* in urine

As in the case of *C. trachomatis*, many of the VOCs with VIPs ≥ 2 were alcohols (Table 1). Among them, 1-tridecanol was the VOC with the highest VIP score (VIP = 3.05) (Table 3). This compound is a long-chain fatty alcohol, and based on the literature, it has been mostly detected in feces as a biomarker of gastrointestinal diseases (Garner et al. 2007). As can be observed, 1-tridecanol is present in the transport medium and is consumed in all the samples, although this decrease is less noticeable in *M.* *genitalium* samples.

Moreover, as occurs in *C. trachomatis*, several C_12_ alcohols (2-dodecanol, 3-dodecanol, 4-dodecanol, and 5-dodecanol) were observed in higher presence in the urine of infected patients (Table 1). As previously mentioned, the dodecanal present in the transport medium could be reduced to the derived alcohols. Other compounds formed by a 12-carbon chain, such as 4,6-dimethyldodecane and dodecyl acetate, were also increased in samples from patients with *M.* *genitalium* infection, possibly derived from dodecanal.

In addition to 4,6-dimethyldodecane, other alkanes such as 4-methyltetradecane and 2-methylpentadecane were more present in the urine of patients with this STI than in the negative ones (Table 1). Alkanes synthesis is not frequent in bacteria, being this pathway identified for the first time in cyanobacteria (Coates et al. 2014). Although the formation mechanism of these alkanes is unknown, their presence in urine could be related to STI caused by *M. genitalium*.

### Volatile biomarkers of the presence of *Neisseria gonorrhoeae* in urine

Among the VOCs with VIPs ≥ 2, 5 were alcohols, as in the other two bacteria in the urine samples. In this case, the VOC with the highest VIP score, and more related to positive urine samples, was 3,4,4-trimethyl-2-cyclohexen-1-one (VIP = 3.10) (Table 3). This ketone was found in the transport medium and increases in all samples with this STI-causing bacteria, being this increase greater in the *N. gonorrhoeae* group.

In addition, among the VOCs with VIP ≥ 2 more related to the presence of *N.* *gonorrhoeae*, as occurs in *C. trachomatis* and *M.* *genitalium*, several C_12_ alcohols (2-dodecanol, 3-dodecanol, 4-dodecanol, and 5-dodecanol) were increased in the urine of infected patients in comparison to the negative ones. Therefore, these three bacteria may have a similar strategy to prevail among the protective microbiota (Table 1).

### Specific volatile biomarkers for the differentiation of the STI-causing bacteria in urine

As can be seen in the scores plot (Fig. 4A), the VOCs with VIPS ≥ 2 allow samples to be grouped according to each bacterium, although with some overlap that could be due to the presence of common VOCs. Thus, VOCs that were not common among bacteria could be considered potential biomarkers of the specific presence of some of the bacteria that cause STIs in urine. In the case of *C. trachomatis*, the urinary VOCs with the greatest potential to be biomarkers would be 2,6-dimethyl-4-heptanone, ethanol, 2-(2-ethoxyethoxy), and 2,5-dimethylbenzaldehyde (in red in Fig. 4B). In contrast, in patients with *M. genitalium* infection, the potential volatile biomarkers in urine were 4-methyltetradecane and 2-methylpentadecane (in blue Fig. 4B), while in *N.* *gonorrhoeae* infection was 3,4,4-trimethyl-2-cyclohexen-1-one (in green Fig. 4B). This report is the first to point to these urinary VOCs as possible volatile biomarkers of STI caused by these bacteria. These results confirm that the 28 VOCs previously identified could be potential volatile biomarkers of the presence of an STI, as well as of a specific STI-causing bacterium.

In conclusion, the identified VOCs made it possible to detect the presence of *C. trachomatis* both in vaginal exudates and urine, with 2,6-dimethyl-4-heptatone being the volatile compound most related to the presence of this bacterium. Despite this, the differentiation of patients with and without this STI was better in urine samples. For that reason, urine samples from patients with other STIs were studied to find volatile markers. Thus, 20 and 9 VOCs were identified as potential biomarkers in the urine of patients with *M. genitalium* and *N. gonorrhoeae*, respectively, making it possible to differentiate infected patients from those without these STIs. Among them, compounds such as 4-methyltetradecane and 2-methylpentadecane were selected as biomarkers of *M. genitalium* infection, or 3,4,4-trimethyl-2-cyclohexen-1-one in *N.* *gonorrhoeae* infection. Moreover, in general, C_12_ alcohols were the major VOC family presented in positive urine samples in all three bacteria, which could indicate the presence of aldehyde reductases in the metabolism of these bacteria.

In short, to contribute to the control of STIs, it is essential to have diagnostic tests that are accessible, fast, easy to perform, and with high sensitivity and specificity. The VOCs identified as potential biomarkers in patients with *C. trachomatis*, *M. genitalium*, and *N. gonorrhoeae* infection could be used in these STIs diagnosis and could be performed in asymptomatic patients, obtaining immediate results that allow establishing a targeted treatment, thus interrupting the chain of transmission, and avoiding new infections.

## Data Availability

All data can be provided by the corresponding author upon request.
